# Cowhage-Induced Itch as an Experimental Model for Pruritus. A Comparative Study with Histamine-Induced Itch

**DOI:** 10.1371/journal.pone.0017786

**Published:** 2011-03-14

**Authors:** Alexandru D. P. Papoiu, Hong Liang Tey, Robert C. Coghill, Hui Wang, Gil Yosipovitch

**Affiliations:** 1 Department of Dermatology, Wake Forest University School of Medicine, Winston-Salem, North Carolina, United States of America; 2 Department of Neurobiology and Anatomy, Wake Forest University School of Medicine, Winston-Salem, North Carolina, United States of America; 3 Department of Regenerative Medicine, Wake Forest University School of Medicine, Winston-Salem, North Carolina, United States of America; Université Paris Descartes, France

## Abstract

**Background:**

Histamine is the prototypical pruritogen used in experimental itch induction. However, in most chronic pruritic diseases, itch is not predominantly mediated by histamine. Cowhage-induced itch, on the other hand, seems more characteristic of itch occurring in chronic pruritic diseases.

**Objectives:**

We tested the validity of cowhage as an itch-inducing agent by contrasting it with the classical itch inducer, histamine, in healthy subjects and atopic dermatitis (AD) patients. We also investigated whether there was a cumulative effect when both agents were combined.

**Methods:**

Fifteen healthy individuals and fifteen AD patients were recruited. Experimental itch induction was performed in eczema-free areas on the volar aspects of the forearm, using different itch inducers: histamine, cowhage and their combination thereof. Itch intensity was assessed continuously for 5.5 minutes after stimulus application using a computer-assisted visual analogue scale (COVAS).

**Results:**

In both healthy and AD subjects, the mean and peak intensity of itch were higher after the application of cowhage compared to histamine, and were higher after the combined application of cowhage and histamine, compared to histamine alone (p<0.0001 in all cases). Itch intensity ratings were not significantly different between healthy and AD subjects for the same itch inducer used; however AD subjects exhibited a prolonged itch response in comparison to healthy subjects ( p<0.001).

**Conclusions:**

Cowhage induced a more intense itch sensation compared to histamine. Cowhage was the dominant factor in itch perception when both pathways were stimulated in the same time. Cowhage-induced itch is a suitable model for the study of itch in AD and other chronic pruritic diseases, and it can serve as a new model for testing antipruritic drugs in humans.

## Introduction

Histamine has been the prototypical pruritogen used in experimental itch induction for many decades [1], [2]. However, in many chronic pruritic conditions, such as atopic dermatitis (AD), the histaminergic pathway does not seem to play the major role since antihistamines are known to be largely ineffective. Some of the characteristics of itch which are common in chronic pruritus, such as itch occurring without a flare and also the mechanically-induced itch, cannot be attributed to the histaminergic pathway.

The rationale of this study was to evaluate and establish a new model of itch in humans, which relates to the PAR-2 pathway. The PAR-2 receptors have been implicated in the pathophysiology of itch and of atopic eczema. Cowhage spicules provide an exogenous route to stimulate PAR-2 receptors in the skin and to elicit itch. Cutaneous application of spicules of the plant cowhage (*Mucuna pruriens var. pruriens*) produces itch without axonal reflex flare [3]–[5]. Cowhage-induced itch is also usually accompanied by a burning and/or pricking sensation [6], [7], which appear to correspond to the nociceptive and burning sensations that accompany itch in atopic dermatitis [8], [9]. Cowhage stimulates nerve fibers distinct from those activated by histamine, and these are polymodal C- neurons that can transmit mechanical and other noxious signals, in addition to itch [10], [11]. The active ingredient of cowhage is mucunain, a cysteine protease, which binds to proteinase-activated receptors-2 and 4 (PAR-2 and PAR-4) [12]. Furthermore, it was previously found that the epidermis and cutaneous nerve fibers of AD patients express elevated levels of PAR-2 [13]. Other endogenous proteases binding to PAR-2, such as cathepsin S, mast cell tryptase and kallikrein [14] may play an important role in mediating pruritus in AD. Therefore, the active ingredient in cowhage, mucunain, acting as an exogenous PAR-2 ligand, may provide a model to study itch in AD and other chronic pruritic diseases.

As cowhage has not been employed in previous clinical or translational studies, we aimed to test the validity of cowhage as an experimental itch model, and contrast it with histamine, in AD patients and healthy controls. We also planned to investigate if there was a summation effect when both agents are administered simultaneously to induce itch.

## Materials and Methods

### Subjects and setting

Fifteen healthy individuals and fifteen AD patients were recruited at the Wake Forest University Health Sciences Department of Dermatology, Winston-Salem, North Carolina, USA. This clinical research involving human participants has been approved by the Internal Review Board of Wake Forest University Heath Sciences. Informed consent has been obtained in all cases and the investigation has been conducted according to the principles expressed in the Declaration of Helsinki. The diagnosis of AD was made using the Hanifin and Rajka criteria [15]. The severity of disease was assessed using the Eczema Area and Severity Index (EASI) [16]. The assessment was performed for all subjects at a screening visit, prior to experimental itch induction, by the same investigator (AP). AD subjects had to present a baseline itch intensity of minimum 3 out of 10 on a Visual Analog Scale (VAS) in order to qualify, and an eczema-free area on the volar aspect of either forearm. The AD group included 8 males and 7 females, average age 32.6±11.2 years (age range 21 to 54), while the healthy group was comprised of 7 males and 8 females, average age 30.9±6.0 years (age range 19 to 41).

Subjects were required to cease all systemic antipruritic medications, including antihistamines, at least 1 week prior to the study. No topical agents were allowed to be applied to their forearms for at least 1 week, but these could be used on other parts of the body.

### Itch induction

Itch stimuli were applied alternatively to the volar aspect of the forearms in the following sequence: histamine (right forearm), cowhage (left forearm), and cowhage and histamine together (right forearm). Between itch inductions, a break was taken to allow previous itch sensations to completely subside. The combination of the two stimuli was administered on the right forearm in an area 10 cm away from the area where histamine was first applied. Histamine and cowhage were delivered on eczema-free areas. A 1% solution of histamine dissolved in 2% methylcellulose gel (Sigma, St. Louis, USA) was delivered using a current of 200 µA through a round iontophoresis electrode, 14 mm in diameter, for 30 seconds (Perimed PF 3826 Perilont Power device; Perimed, Sweden) as we previously reported [2]. Itch intensity was assessed continuously for 5.5 minutes subsequently.

After the itch sensation from histamine iontophoresis had completely subsided, an eczema-free area on the other forearm was used for cowhage application. A number of 40 to 45 cowhage spicules were counted under the microscope, picked-up by a microtweezer and were applied within a 4 cm^2^ circular area on the skin. The spicules were gently rubbed for 45 seconds onto the subjects' skin with a circular motion to facilitate contact; a cotton cloth was used to demarcate the area to prevent any stray spicules from stimulating surrounding skin. (A previous study had reported that 1 spicule was sufficient to induce a significant itch, and furthermore, there was no difference in sensation intensity when 1 or 7 spicules were inserted over a small area [6]). Subjects were instructed to ignore the initial stinging or pricking sensations and rate only the itch sensation *per se*. After 5.5 minutes, during which itch intensity was continuously reported, the spicules were removed using adhesive tapes (3M, St.Paul, MN)

When itch sensation induced by cowhage completely subsided, an area on the contralateral forearm (10 cm away from the area that was used for histamine's single application previously) was chosen for application of both histamine and cowhage. Histamine was administered for 30 seconds by iontophoresis as described above. Immediately thereafter (approximately 1 minute later), cowhage was applied to an adjacent site (1 cm away). Itch intensity was assessed continuously for another 5.5 minutes. Subjects were instructed to rate the itch sensation from both stimuli together after the application of cowhage was finalized.

### Quantitative psychophysical assessment

Subjects used a computerized visual analogue scale (COVAS, Medoc, Ramat-Yishai, Israel) for continuous reporting of itch intensity for a duration of 5.5 minutes. The COVAS allows rating of itch intensity on a 100 mm scale that ranges from “no itch” at one end to “unbearable itch” at the other. The subjects can slide an indicator between the two ends to reflect the intensity of their sensations and the values were continuously registered by a computer throughout the experimental period. Visual analog scales have long been used to assess various sensations and have been shown to exhibit ratio-scale properties for psychophysical ratings [17] (Price et al. 1994) and to be more reproducible than verbal descriptor scales [18] (Rosier et al. 2002). Continuous ratings over time acquired via computerized VAS closely mirror post-stimulus retrospective ratings [19] (Koyama et al. 2004). We have adapted the VAS and the COVAS use for ratings of itch perception and have demonstrated that these scales are 1) sensitive to manipulations which can reduce itch, 2) capable of assessing changes in itch intensity over time, and 3) able to distinguish differences between acute and chronic itch [20], [21].

#### Statistical analysis

COVAS data were sampled at 9 Hz. Custom written programs in IDL (ITT Visual Information Solutions, Boulder, CO) were used to extract the peak itch rating as well as ratings at 30 seconds intervals. These programs were also used to calculate the average itch over time, and to calculate group averages.

Using JMP software we examined the entire perception (intensity ratings) curves for the full duration, identified the peak value and calculated the means.Peak and mean itch intensity ratings were analyzed using a two factor analysis of variance (ANOVA, JMP Software: SAS, Cary, NC) assessing effects of itch stimulus (cowhage, histamine, and cowhage+histamine) within subjects, and effects of disease state (healthy vs. atopic patients) across subjects, as well as the interaction between these factors. Orthogonal comparisons were used to determine differences between pairs of itch stimuli.

Time courses of itch were analyzed at 30 seconds intervals using a three factor analysis of variance (ANOVA), assessing the effects of time and itch stimulus within subjects, and the effects of disease status across subjects. As above, orthogonal comparisons were used to determine differences between pairs of itch stimuli, compared to the reference value, set for time point = 30 seconds.

Separate linear regression analyses were employed for each itch stimulus in order to determine if the evoked itch intensity was significantly influenced by the severity of AD, as evaluated by. EASI scores.

## Results

### Differences in mean and peak itch intensity ratings for itch induced by cowhage, histamine and their combination

**Figure 1:**
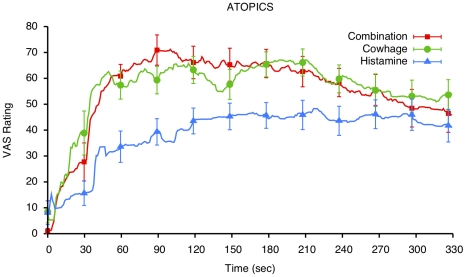
Time course of continuous itch intensity ratings (on a visual analog scale of 0 to 100 mm) in atopic dermatitis subjects, following itch induction with histamine, cowhage and their combination. A significant difference in time course and in the magnitude of response is observed between cowhage and histamine-induced itch (p<0.0001).

**Figure 2:**
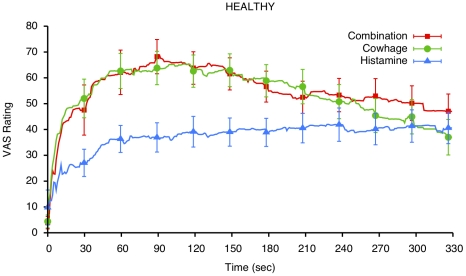
Time course of continuous itch intensity ratings (on a visual analog scale of 0 to 100 mm) in healthy subjects, following itch induction with histamine, cowhage and their combination. A significant difference in time course and in the magnitude of response is apparent between cowhage and histamine-induced itch (p<0.0001).

Itch intensity measured by COVAS ratings over time in AD patients and healthy subjects are shown in figures 1 and 2. In both groups, the mean and peak COVAS ratings were significantly influenced by the itch stimulus (main effect of stimulus: mean rating p<0.0001, peak rating: p<0.0001). Both peak and mean itch ratings were higher following cowhage application versus following histamine application (both p<0.0001), but were not significantly different between the combined application of cowhage and histamine, compared to cowhage alone (contrast of mean ratings: p = 0.89; for peak ratings: p = 0.29). Although AD patients appeared more sensitive to all stimuli, there was no significant main effect of disease status on itch ratings (mean rating: p = 0.77; peak rating: p = 0.36).

### Continuous itch response curves reveal a different pattern of variation over time between AD and healthy subjects

Analysis of the time course of perceived itch intensity from continuous ratings revealed that itch intensity varied significantly across (over) time (analyzed as a main effect time: p<0.0001, Fig. 1, 2). Ratings gradually rose following the application of the itch stimuli, and then, after reaching a peak, tapered down slowly over time. In healthy participants, itch increased faster (following a steeper slope) and started to subside quicker than in atopic dermatitis patients. This pattern of time dependency was different in AD patients vs. healthy subjects (interaction of time×disease status, p = 0.0025). AD patients exhibited a slower onset of itch than healthy subjects; also, their itch decreased very slowly after it reached peak values. As in the case of the peak and mean (average intensity) ratings, a significant main effect of itch stimulus was detected in the time course data (p<0.0001), and this effect did not vary with disease status (drug×disease status interaction: p = 0.91).

### The Relationship between EASI scores and perceived itch intensity ratings in AD subjects

For histamine-induced itch, the mean COVAS ratings were positively correlated to the EASI scores (r^2^ = 0.32, p = 0.02), and the peak COVAS ratings also were positively correlated to the EASI scores (r^2^ = 0.54, p = 0.0016). The COVAS ratings for cowhage-induced itch and for the itch induced by cowhage and histamine in combination showed no linear relationship with disease severity as evaluated by EASI scores (Table 1).

**Table 1:** EASI scores of the severity of disease in atopic dermatitis subjects included in the study.

Sbj. #	EASI score	Age	Gender
1	15.0	36	m
2	3.8	25	m
3	12.2	22	f
4	3.8	30	f
5	1.1	24	f
6	0.8	54	f
7	12.9	20	f
8	5.2	40	m
9	3.0	21	f
10	9.3	34	m
11	22.5	41	m
12	2.3	24	f
13	17.3	46	m
14	49.0	49	m
15	16.2	23	m
AVG	11.6	32.6	

## Discussion

Histamine is the classic, best known mediator of itch which has been used as the standard experimental pruritogen in numerous studies in the past decades. However, a novel, distinct pathway for itch transmission, which is stimulated by cowhage has been elucidated in recent years. The spicules of this tropical plant release upon skin contact a cysteine protease, mucunain, that binds to PAR2/4 receptors [12] which leads to stimulation of cutaneous polymodal C-fibers [10]; this pathway is distinct from the neuronal circuits transmitting histamine itch, and synapses in a separate subset of spinothalamic secondary neurons [22]. In chronic pruritic diseases of which AD is the most common, the histaminergic pathway does not appear to be the most relevant circuit (antihistamines are ineffective for chronic itch relief). However, cowhage induces an itch associated with burning and pricking sensations, commonly observed in AD, and it works via PAR-2 [12] receptors, which display an increased expression in atopic skin. These findings support the hypothesis that PAR-2 mediated itch pathway may represent the relevant pathway predominantly stimulated in AD.

**Figure 3:**
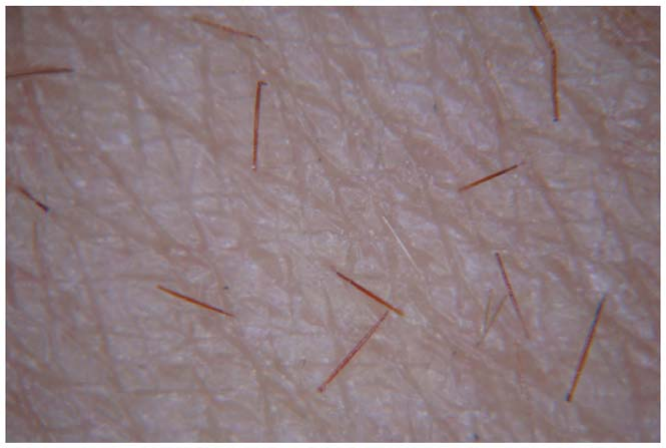
An image of cowhage spicules inserted into the skin by gentle rubbing, under polarizing light at 5× magnification.

This study showed that the response to cowhage was reproducible, reliable and consistent in both healthy and AD subjects. We found that a rather minimal dose of cowhage, approximately 45 spicules, equivalent to 45 µg of material (maximally containing about 90 ng of mucunain) induced a more intense itch sensation compared to histamine, at a dose of 6 mg histamine (base) that was contained in the chamber of the drug delivery electrode. Delivery experiments revealed that the number of cowhage spicules inserted upon rubbing, as examined under regular or polarizing light by magnifying lens at 5× magnification (Fig. 3), represents about a third of the total spicules applied, therefore the actual dose of mucunain deliverable by spicules can be estimated at 15–30 ng.

The co-stimulation of histaminergic and cowhage-itch pathways did not results in a summation of itch intensity. The combination of the stimuli induced an itch that was much more intense than using histamine alone, but it was not significantly more intense compared to itch induced by cowhage alone. This suggests that the major contributor of perceived itch sensation is cowhage, when both pathways are stimulated concurrently.

We propose that cowhage could be used a suitable model to induce experimental itch. Cowhage-itch more closely resembles the characteristics of pruritus in chronic conditions and induces a more intense sensation compared to histamine. Cowhage-induced itch appears as the dominant component of itch perception when these two pathways are stimulated simultaneously. From a therapeutic standpoint, drugs capable to inhibit cowhage itch pathway appear promising to pursue for the treatment of chronic pruritus. Cowhage induce itch could offer a new paradigm to test antipruritics in humans.

We also investigated the potential correlation of the EASI scores, a validated, objective measure of the severity of atopic eczema [16], with the subjective perception of itch intensity in AD subjects. Interestingly, ratings of histamine induced itch were correlated to the EASI scores, while the perceived intensity of cowhage induced itch was not. Although we do not have a definitive explanation for the lack of correlation between EASI scores and the perception of itch induced with cowhage, we note note that clinical features such as lichenification, excoriations, pigmentation and disease extension on body areas (included in this scoring) may have a limited ability to reflect the subjective expression of itch severity in patients [23]. Histamine induced itch causes a neurogenic inflammation, therefore the mechanisms underlying the inflammatory processes involved in the pathophysiological manifestation of atopic dermatitis (factored into the EASI score) may share a common link with the histamine induced response, while the itch induced via PAR-2 pathway may not, since it does not evoke a neurogenic response [10].

Previous studies have shown that AD patients exhibited higher intensity ratings for histamine induced itch than in healthy subjects, suggesting that AD subjects present a hypersensitization of the itch response [20], [24]. In this study, we found only a trend for a higher intensity of itch perception in AD subjects. However, when analyzing itch intensity fluctuation over time, AD patients displayed a significantly prolonged itch response compared to healthy subjects for both stimuli, supporting the notion that AD involves a neuronal hypersensitization at peripheral and/or central levels, in similarity with chronic pain [25].

### Conclusion

Cowhage induced a much more intense itch sensation compared to histamine and it was the dominant player in itch induction when both pathways were activated at the same time. Cowhage-induced itch could serve as a suitable model for the study of itch in AD and other chronic itch diseases.
